# Central Retinal Artery Occlusion Secondary to Patent Foramen Ovale: The Unexpected Journey of a Paradoxical Embolus

**DOI:** 10.7759/cureus.9496

**Published:** 2020-07-31

**Authors:** Nikolaos Sabanis, Georgios Zagkotsis, Vasileios D Krikos, Eleni Paschou, Angelos Tapanlis

**Affiliations:** 1 Department of Nephrology, General Hospital of Livadeia, Livadeia, GRC; 2 Department of Cardiology, Private Medical Office, Livadeia, GRC; 3 Department of Family Medicine, Medical Unit of Saint George, Livadeia, GRC; 4 Department of Emergency Medicine, General Hospital of Livadeia, Livadeia, GRC

**Keywords:** central retinal artery occlusion, patent foramen ovale, metaphlebitic syndrome

## Abstract

Central retinal artery occlusion (CRAO) represents one of the most devastating ophthalmic emergencies, since the inner two-thirds of the retina lose their blood supply. The acute obstruction of the central retinal artery is characterized by severe, sudden and unilateral painless visual loss and usually occurs secondary to an embolus of cardiovascular origin. A paradoxical thromboembolic event of the central retinal artery through patent foramen ovale is an exceptionally unusual clinical entity as well as a great diagnostic challenge since the source of initial thrombus formation requires extensive investigation. Herein, we aim to describe a patient with no significant comorbidities who experienced a paradoxical thromboembolic episode of central retinal artery associated with patent foramen ovale.

## Introduction

Central retinal artery occlusion represents the ophthalmic analogue of ischemic stroke and is typically manifested with sudden, severe and painless loss of vision [1]. However, some patients experience transient visual loss due to an impermanent embolic episode which is also known as amaurosis fungax and corresponding to transient ischemic stroke [2]. Therefore, when CRAO occurs, a severe loss of vision is expected with the exception of cases having an unaffected cilioretinal artery that undertakes the blood supply of the retina and guarantees the restoration of central vision [3].

CRAO is usually the result of an embolus, either of cardiac origin or dislodged from an unstable atherosclerotic plaque of carotid bifurcation. Subsequently, the most common risk factors for CRAO include carotid atherosclerosis, cardiac valvular disease and cardiac arrhythmias, mainly atrial fibrillation [1]. Congenital cardiac anomalies, such as patent foramen ovale (PFO) may be the source of a paradoxical embolic event, although the majority of patients with PFO follow an uneventful and silent clinical course [4].

The most important and well-documented clinical manifestation of PFO is the cryptogenic ischemic stroke due to a paradoxical embolus. CRAO or branch retinal artery occlusion (BRAO) associated with PFO represents an unexpected site of systemic paradoxical embolism that requires high index of clinical suspicion and meticulous laboratory investigation in order to unravel the concealed initial source of thrombus generation [5].

Herein, we aim to present a middle-aged male patient who experienced an episode of severe, painless and unilateral visual loss due to CRAO associated with PFO. We also discuss the clinical complexity of such a diagnosis as well as we briefly review the current literature regarding similar published case reports.

## Case presentation

A 62-year-old Caucasian male, with no previous ocular or neurological symptoms, experienced a sudden and painless loss of vision in the left eye while swimming. He had no significant past medical history apart from an episode of deep vein thrombosis of the left lower extremity four years ago, provoked by a non-displaced fracture of the tibia after a motor vehicle accident. He also reported a previous history of hospitalization due to an episode of acute left-sided loin pain associated with macroscopic haematuria at the age of 60 years. Then the patient had been thoroughly investigated and no specific diagnosis of renal infarct of unknown origin had been made. Otherwise, he did not take any regular medication and he neither smoked tobacco nor drank alcohol.

On initial examination, he was afebrile and his pulse rate was 82/min and regular. His blood pressure was 130/85 mmHg and there were no abnormalities to find in the cardiovascular or respiratory systems. No carotid or over the umbilicus bruits were audible and pedal pulses were palpable. Physical examination was otherwise normal apart from a clearly swollen left lower extremity accompanied with engorged superficial veins, hyperpigmentation and trophic skin changes. On palpation, there was no tenderness of his left calf muscles and no venous ulcerations were observed. As regards neurological examination it was also unremarkable including the muscle tone, reflexes, coordination and sensation as well as power, in all muscle groups. 

**Figure 1:**
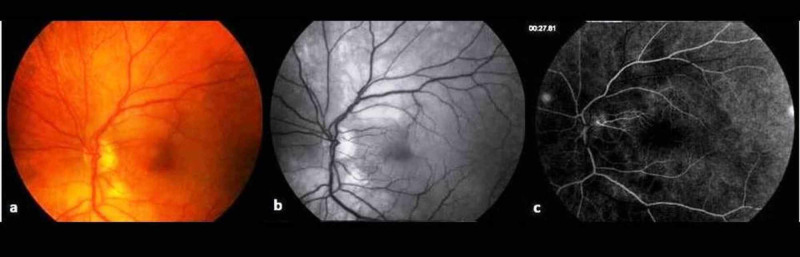
(A) Fundoscopic examination (B) Fundus autofluorescence (FAF),  (C) Fluorescein angiography (FA)

Afterwards, the patient was immediately evaluated on the basis of fundoscopic examination that revealed left retinal whitening, macular edema and cotton-wool spots compatible with CRAO (Figure 1A). The above findings were also evident in the fundus autofluorescence (FAF) imaging (Figure 1B). Retinal whitening corresponded to ischemic damage to the inner half of the retina with opacification of the retinal nerve fiber and ganglion cell layers due to cessation of axoplasmic transport. The retinal periphery appeared normal. Fluorescein angiography (FA) showed a delayed arterial filling and a patent cilioretinal artery, derived from the posterior ciliary circulation, supplying part of the papillomacular bundle (Figure 1C). At the arteriovenous phase, the retinal vascular wall exhibited fluorescein leakage and there was a focal hypofluorescence in the region of the cotton wool spot. The optic disc appeared normal during the early and late phases.

Once CRAO with sparing of cilioretinal artery diagnosis had been made, a meticulous investigation was performed in order to unwind the thread of this unanticipated thromboembolic event.

Routine hematological and biochemical investigations were unremarkable barring the slightly elevated creatinine and urea levels, up to 1.6 mg/dL and 60 mg/dL respectively, but accordant with a prolonged history of chronic kidney disease stage III with GFR estimated at 46ml/min/1.73m2. Inflammatory markers (C- Reactive Protein and Erythrocyte Sedimentation Rate) and clotting parameters including d-dimers and fibrinogen levels were within normal values. Further investigation for autoimmune diseases, occult infectious conditions and thrombophilic disorders were also negative. More specifically, thrombophilia testing was performed including factor V Leiden, prothrombin gene mutation, antithrombin III, protein C and protein S, lupus anticoagulant screen as well as antinuclear antibodies, anti-dsDNA antibodies, anti-cardiolipin antibodies (ACA) and anti-beta 2 glycoprotein antibodies (anti-β2GPI).

Regarding radiological examination, a renal ultrasound confirmed the previously mentioned asymmetrical kidney size and showed a small as well as echogenic left kidney, consistent with the past history of renal infarction without apparent cause. Carotid Doppler ultrasound did not identify any extracranial vulnerable atherosclerotic plaque potentially at risk for disruption and thromboembolic ischemic retinal event. Similarly, abdominal aorta ultrasound imaging and ultrasonographic assessment of renal arteries did not depict any atherosclerotic lesions that could be possibly the source of the reported renal vaso-occlusive event.

Venous ultrasound imaging unveiled no relapse of the previously recorded deep vein thrombosis although the diagnosis of post-thrombotic syndrome was made on the basis of post-thrombotic popliteal valve incompetence accompanied with significant reflex at the same level. Residual thrombi were also recognized mainly in the left popliteal vein and partially recanalization of both popliteal and gastrocnemius veins was described. Residual thrombi in left popliteal vein raised the question of whether a paradoxical embolus could explain not only the CRAO event but also the past embolic event in renal artery circulatory system.

**Figure 2:**
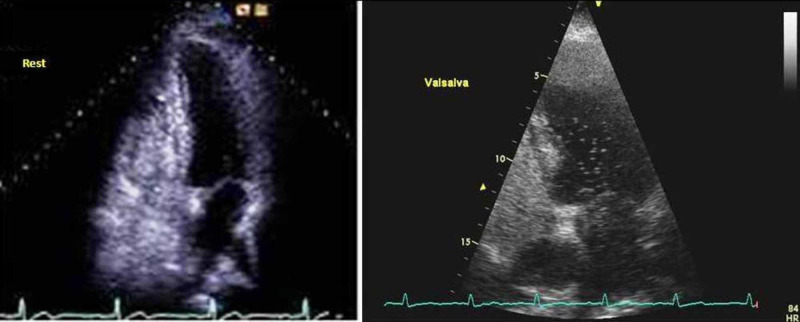
Bubble contrast transthorachic echocardiography a) on rest, b) during Valsava maneuver

With the completion of these steps, a meticulous cardiologic examination was undertaken and included 48-hour Holter monitoring and transthoracic echocardiography followed by bubble contrast echocardiography testing. Holter monitoring results excluded the possibility that CRAO event could be correlated with an obscured supraventricular arrhythmia, predominantly an episode of paroxysmal and symptomless atrial fibrillation. Transthoracic echocardiography examination also eliminated the option that the embolic event originated from a cardiac valvular defect. Surprisingly, bubble contrast echocardiography testing revealed more than 25 saline microbubbles in the left atrium of the heart at the first three cardiac cycles while the patient was asked to perform a Valsava maneuver (Figure 2). On the basis of these findings, the presence of PFO was confirmed, since the Valsava maneuver transiently raised the pressure in the right side of the heart resulting in the entering of saline microbubbles in the left atrium through PFO.

On combining this finding with the presence of residual thrombi in the left popliteal vein, we reasonably conclude that a paradoxical thromboembolic event was the cause not only of CRAO but also the previously recorded event of systemic embolism in the left kidney. It is also noteworthy, that the patient reported that in both cases the embolic events occurred while performing strenuous underwater swimming, which probably included Valsava maneuvers.

Afterwards, the patient received oral anticoagulant therapy with apixaban at a dose of 5 mg twice daily as well as strict instructions concerning post-phlebitic syndrome, that included elastic compression stockings, exercise and lifestyle modifications, given that he refused to undergo any surgical or endovascular intervention. Over and above that, the patient remains symptomless one year after the last thromboembolic event and on regular follow-up, he seems to preserve satisfactory central vision. 

## Discussion

CRAO is an ocular vascular occlusive disorder with potentially devastating repercussions regarding visual acuity since the inner two-thirds of the retina lose their blood supply. Therefore, if complete CRAO occurs, a retinal infarction will be installed within 12-15 min while the inner retina is critically dependent on the end-central retinal artery circulation and probably more vulnerable to oxygen deprivation than brain [6].

Almost similarly to ischemic stroke, retinal stroke after CRAO lasting for about four hours results in acute, irreversible, unilateral and painless vision loss [7]. Noteworthy, some CRAO patients unravel also a history of transient vision loss due to an ephemeral and incomplete CRAO, also known as amaurosis fugax, which correspondingly to transient ischemic stroke may be a harbinger of complete CRAO [2].

However, a window of opportunity for the central visual acuity may still exist if an unaffected cilioretinal artery is present. Cilioretinal artery, a common anatomic variant of ophthalmic circulation, is present in 15-30% of the general population and in CRAO, patients seem to have a protective effect on macular retina and a major impact on visual outcome [3].

Hence, CRAO is a rare vaso-occlusive event, with incidence estimated to be 1.90 per 100.000 among the United States white population [8] and usually shares pathophysiology with acute ischemic stroke, particularly valvular disease, smoking, atrial fibrillation/dysrhythmias as well as atherosclerotic carotid disease [1]. The most common cause of CRAO is thought to be either a cardioembolic event or an artery-artery embolism, principally originated from the ipsilateral carotid artery. Carotid artery disease can provoke CRAO through embolism from an unstable atherosclerotic plaque or in the case of significant internal carotid artery stenosis by markedly reduced ocular blood flow [9]. Less commonly, CRAO has been associated with a plethora of systemic diseases and miscellaneous conditions, including collagen vascular disorders, infections, hemoglobinopathies such as sickle cell disease, myeloproliferative neoplasms mainly polycythemia vera, thrombophilic conditions, oral contraceptives, increased intraocular pressure due to glaucoma and iatrogenic causes like the use of injectable hyaluronic acid fillers.

However, giant cell arteritis should be always considered in elderly patients since ocular symptoms in patients with ocular involvement include visual loss of varying severity and amaurosis fugax due to CRAO, cilioretinal artery occlusion or even arteritic ischemic optic neuropathy [10]. On the other side, in selected CRAO patients younger than 40 years, thrombophilia screening may yield positive results in a high percentage of cases [11]. Assuredly, in young patients with retinal arterial occlusion, cardiac valvular disease is assumed to be the most commonly identified etiologic factor in parallel to various associated conditions leading to a hypercoagulable state that render CRAO a complex event of multifactorial etiology via different mechanisms [12]. In the same context, PFO should be also considered in the young or middle-aged CRAO patients since PFO represents the most commonly recognized congenital interatrial cardiac defect and potentially a potent source of recurrent paradoxical emboli [13]. In particular, the prevalence of PFO in the healthy adult population has been estimated to range from 15-33% and 15-25% on autopsy and echocardiographic studies respectively [4, 14]. 

As regards to our patient and within the above framework, we have to underline that once the diagnosis of CRAO with patent cilioretinal artery had been made, a meticulous and multifarious approach should be performed in order to unravel the source of the embolic event. The inability to identify common atherosclerotic risk factors such as diabetes mellitus, hypertension, dyslipidemia and smoking or even more significant atherosclerotic lesions despite a thorough investigation was initially an intriguing datum. With this in mind, a stepwise and vigilant cardiologic evaluation revealed eventually a PFO under certain conditions, particularly Valsava maneuvers during bubble saline contrast with transthoracic echocardiography.

On the basis of this finding and taking into account the presence of residual thrombi in the left popliteal vein due to post-thrombotic syndrome, we reasonably presume that CRAO was the result of a paradoxical embolus through PFO. Certainly, other coexisting risk factors for recurrent arterial thromboembolic events such as mitral or aortic valvular diseases, unidentified atrial fibrillation and underlying thrombophilic conditions, mainly antiphospholipid syndrome were excluded. The possibilities of giant cell arteritis as well as various infectious diseases associated with CRAO were also evaluated relying on their distinct clinical and laboratory characteristics and were negative. 

At the same time, we also postulate that the previously reported renal infarct of unknown origin was similarly the result of another paradoxical embolus, originated from the left popliteal or gastrocnemius veins through PFO. Outstandingly, our patient described that both events developed while performing vigorous underwater swimming which plausibly included Valsava maneuvers. In favor of this, it is a widely known fact that arterialization of gas bubbles seems to be more frequent than usually presumed in recreational divers in the light of the high prevalence of venous gas bubbles even after diving in shallow water and the presence of a cardiac right-to-left shunt (PFO) in a quarter of the population [15].

The frequency and significance of PFO in association with ocular circulatory disturbances were evaluated in forty patients with acute arterial occlusions of the posterior bulb segment through transthoracic and transesophageal echocardiography by Steuber and colleagues [16]. PFO was identified in nine patients and two of them showed that the potential source of paradoxical embolism was phlebothrombosis in their clinical history. Similarly to our case, cardiovascular risk factors were not prevalent in the group with PFO and both groups had a mean age of approximately 60 years. The embolic potential of PFO was also exemplified in the work undertaken by Yuan and referred to a 53-year-old man who diagnosed with embolic events including transient ischemia attack, retinal artery occlusion and left kidney infarct accompanying mitral valve thrombus associated with a PFO [17].

Certainly, the emerging association between undiagnosed PFO and retinal artery occlusion has been predominantly reported in young patients. An example of this is the report of a 35-year-old man with branch retinal artery occlusion (BRAO) and concomitant PFO, which was firstly diagnosed during the evaluation of BRAO, by Chatziralli and colleagues [18]. In a similar case, Shoebii et al. reported a 29-year-old female patient with BRAO secondary to PFO and underlined the fact that intracardiac right-to-left shunting through a PFO, accentuated by Valsalva maneuver, may predispose to embolic events while the source of initial thrombosis remains unknown [5]. In the same context, Ho and Spaide, reported a case of a boy with CRAO and cilioretinal sparing secondary to PFO soon after a fractured left clavicle and speculated that although their patient did not show any clinical signs of deep venous thrombosis, his dehydration and immobility post-injury might have contributed to a relative thrombophilia with a thrombus embolizing into the arterial system via the patent foramen ovale [19]. Overall, the above cases support the view that paradoxical thromboembolic event of central or branch retinal artery through PFO is an exceptionally rare clinical entity as well as a great diagnostic challenge, since the source of initial thrombus formation may be concealed despite extensive investigation.

Hence, it could be conceivably argued that retinal artery occlusion in an otherwise healthy adult patient, should always consider the PFO as a differential that requires meticulous cardiologic examination. Despite the fact that transesophageal echocardiography (TEE) is a potentially useful and effective tool for detecting possible sources of retinal artery emboli [20], in our patient, bubble contrast echocardiography testing revealed the presence of PFO while he was asked to perform a Valsava maneuver in real time. Following that and taking into consideration the presence of residual thrombi in the left popliteal vein, as well as the previously recorded event of systemic embolism in renal circulation, we recommended a percutaneous closure of PFO. Contrary to our expectations, the patient refused any interventional procedure and eventually received antithrombotic treatment with a direct oral anticoagulant.

## Conclusions

Patent foramen ovale (PFO) is a common congenital intracardiac defect that can cause right-to-left shunting under certain conditions and may predispose individuals to paradoxical embolic events from the venous to arterial district. Rarely, PFO has been correlated to central retinal artery occlusion and branch retinal artery occlusion with visual sequelae not only in young but also in advanced-aged patients at low atherosclerosis or cardioembolic risk, concomitant with a propensity for deep vein thrombosis. 
